# The complete chloroplast genome and phylogenetic analysis of *Piptanthus concolor* Craib (Leguminosae)

**DOI:** 10.1080/23802359.2021.1923410

**Published:** 2021-07-14

**Authors:** Hai-ling Li, Min Yang, Nong Zhou

**Affiliations:** a College of Pharmacy, Dali University, Dali, China; b College of Food and Biology Engineering, The Chongqing Engineering Laboratory for Green Cultivation and Deep Processing of the Three Gorges Reservoir Area's Medicinal Herbs, Chongqing Three Gorges University, Chongqing, China

**Keywords:** *Piptanthus concolor*, chloroplast genome, Illumina sequencing, phylogenetic analysis

## Abstract

In this study, we used high-throughput sequencing technology to sequence and functionally annotate the chloroplast genome of *Piptanthus concolor*. The total length of *P. concolor* chloroplast genome is 152,115 bp with the typical quadripartite structure. It contains two inverted repeats (IRa and IRb) of 26,233 bp each, which are separated by a large single-copy (LSC) region of 82,024 bp and a small single-copy (SSC) region of 17,625 bp. The cp genome contained 111 genes, including 77 protein-coding genes, 30 tRNA genes, and 4 rRNA genes. Phylogenetic analysis based on the whole chloroplast genome showed that *P. concolor* was closely related to *Ammopiptanthus*.

*Piptanthus* Sweet is a small genus in the family Leguminosae, three species and two forms of which have been found in China. *Piptanthus concolor* Craib is widely distributed in the region from the Himalaya to the Hengduan Mountains (Sun 2002). The seeds of *P. concolor* are used as Tibetan folk medicine (Liu and Jia 1991). Eleven isoflavonoids were isolated and identiffied from stem of *P. concolor* (kou et al. 2012). karyotype analysis of *P. concolor* was performed with fluorescence in situ hybridization(FISH) (Luo et al. 2017). However, there are rare reports about the chloroplast (cp) genome information of *Piptanthus*. In this study, we determined the complete chloroplast genome of *P. concolor* based on high throughput sequencing technology, which will provide bioinformatics data for the phylogeny of *Piptanthus* genus and further research on Leguminosae.

The fresh leaves of *P. concolor* were collected from Dali, Yunnan province, China (100°6’43.1′’E, 25°50’46.07′’N). The voucher specimen (NO: ZDQ17066) was deposited at the Herbarium of Medicinal Plants and Crude Drugs of the College of Pharmacy, Dali University. Total genomic DNA was extracted from the fresh leaves using a modified CTAB method (Doyle 1987; Yang et al. 2014). Genome sequencing was performed using Illumina Hiseq 2500 (Novogene, Tianjing, China) platform with pair-end (2 × 300bp) library. The raw data have filtered using Trimmomatic version 0.32 with default settings (Bolger et al. 2014). Then the filtered reads were assembled into circular contigs using GetOrganelle (Jin et al. 2020), with the complete chloroplast genome of *Ammopiptanthus mongolicus* as the reference (GenBank accession no. KY034453). Finally, the chloroplast genomes were annotated using Geneious R11.0.2 (Kearse et al. 2012), and corrected manually for start and stop codons. The complete chloroplast genome sequence of *P. concolor* was submitted to GenBank with the accession number of MT627346.

The complete chloroplast genome of *P. concolor* is 152,115 bp in length with the typical quadripartite structure, which consists of a large single copy-region (LSC, 82,024 bp), a small single copy-region (SSC, 17,625 bp), and a pair of inverted repeat regions (IRa and IRb, 26,233 bp). The full-sequence GC content of the chloroplast genome was 36.6%. 111 chloroplast genes were successfully annotated, including 4 rRNA genes, 30 tRNA genes and 77 protein-coding genes. 12 genes contain introns, among which 10 genes have only one intron and 2 genes (*ycf3* and *clpP*) have two introns．

In order to determine the phylogenetic position of *P. concolor*, we selected 32 chloroplast genome sequences from NCBI. All sequences were aligned using program MAFFT v.7.149 (Katoh and Standley 2013). and a neighbour-joining (NJ) phylogenetic tree was constructed by MEGA v.7.0.26 (Kumar et al. 2016) with 1000 bootstrap replicates. The phylogenetic analysis showed that *P. concolor* was closely related to *Ammopiptanthus* (Figure 1).

**Figure 1.**
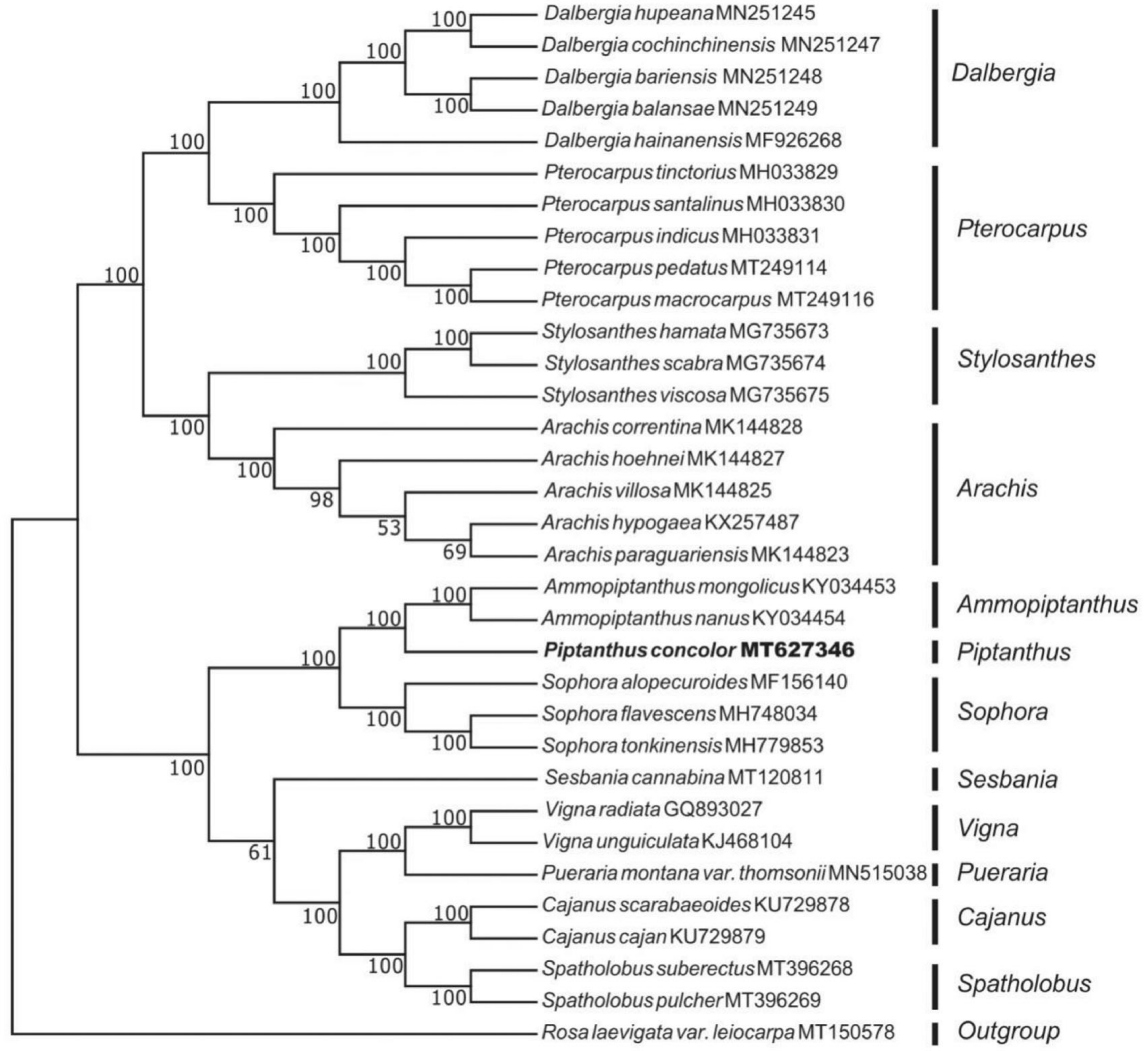
Neighbor-joining (NJ) tree of 32 species from the family Leguminosae based on the complete chloroplast sequences using *Rosa laevigata var. leiocarpa* as an outgroup.

## Data Availability

The genome sequence date that support the findings of this study are openly available in GenBank of NCBI at (https://www.ncbi.nlm.nih.gov/) under the accession no. MT627346.
